# *De Novo* Genome Sequence of “*Candidatus* Liberibacter solanacearum” from a Single Potato Psyllid in California

**DOI:** 10.1128/genomeA.01500-15

**Published:** 2015-12-17

**Authors:** F. Wu, X. Deng, G. Liang, C. Wallis, J. T. Trumble, S. Prager, J. Chen

**Affiliations:** a Department of Plant Pathology, Laboratory of Insect Ecology, South China Agricultural University, Guangzhou, Guangdong, China; b San Joaquín Valley Agricultural Sciences Center, USDA-ARS, Parlier, California, USA; c Department of Entomology, University of California, Riverside, Riverside, California, USA

## Abstract

The draft genome sequence of “*Candidatus* Liberibacter solanacearum” strain RSTM from a potato psyllid (*Bactericera cockerelli*) in California is reported here. The RSTM strain has a genome size of 1,286,787 bp, a G+C content of 35.1%, 1,211 predicted open reading frames (ORFs), and 43 RNA genes.

## GENOME ANNOUNCEMENT

“*Candidatus* Liberibacter solanacearum,” an unculturable alphaproteobacterium, inhabits both potato psyllid *Bactericera cockerelli* (Šulc) (Hemiptera: Triozidae) and solanaceous plants. Infection of the bacterium is associated with potato zebra chip (ZC), a recently emerging potato disease in North America and elsewhere (1, 2). The bacterium is primarily vectored between solanaceous plants by potato psyllids. In California, the bacterium was first detected in the Lancaster area under the name of “*Candidatus* Liberibacter psyllaurous” (3)”, a synonym of “*Ca.* Liberibacter solanacearum.” “*Ca.* Liberibacter solanacearum” is mostly characterized through genomic approaches, since it cannot be cultured *in vitro*. We previously published the draft genome of “*Ca.* Liberibacter solanacearum” strain R1, which originated from California and was maintained in tomato plants grown in a greenhouse (4). Here, we report a draft genome sequence of “*Ca.* Liberibacter solanacearum” strain RSTM isolated from a potato psyllid in California.

“*Ca.* Liberibacter solanacearum” strain RSTM was originally collected from psyllids at the University of California, South Coast Research and Extension Center in Irvine, CA, and maintained in a greenhouse at University of California, Riverside, Riverside, CA. Potato psyllids free of “*Ca.* Liberibacter solanacearum” were fed on the infected tomato plants. Adult psyllids were collected. The DNA of an individual psyllid was extracted using the DNeasy blood and tissue kit (Qiagen, Valencia, CA). Infection of “*Ca.* Liberibacter solanacearum” was monitored by use of the real-time PCR method of Li et al. (5). DNA from a single psyllid sample (threshold cycle [*C_T_*], 17.2) was selected and amplified through illustra GenomiPhi version 2 DNA amplification kits (GE Healthcare, Inc., Waukesha, WI, USA). The amplified DNA was sequenced using the Illumina MiSeq format (Illumina, San Diego, CA).

A total of 3.82 × 10^7^ reads with a mean of 251 bp per read were generated from MiSeq sequencing. *De novo* assembly was performed with CLC Genomics Workbench 7.5. Among the 97,229 large contigs (≥1,000 bp) generated, 26 contigs ranging from 1,125 bp to 342,256 bp, with an average coverage of 1,543×, were identified as being associated with “*Ca.* Liberibacter solanacearum” by standalone BLASTn (version 2.2.30) (6), using the genome sequence of “*Ca.* Liberibacter solanacearum” strain R1 (4) as a reference. The contigs were extracted using a Perl script and constituted the draft genome of “*Ca.* Liberibacter solanacearum” strain RSTM. Annotation was performed by the RAST server (http://rast.nmpdr.org/) (7). The RSTM genome comprises 1,286,787 bp, with a G+C content of 35.1%, 1,211 predicted open reading frames (ORFs), and 43 RNAs.

### Nucleotide sequence accession numbers.

This draft genome sequence has been deposited in DDBJ/EMBL/GenBank under the accession no. LLVZ00000000. The version described in this manuscript is the first version, LLVZ01000000.
